# CRISPR/Cas System-Based Biosensors

**DOI:** 10.3390/bios16020117

**Published:** 2026-02-10

**Authors:** Xingjie Hu, Jing Su, Shiping Song

**Affiliations:** 1 State Key Laboratory of Oncogenes and Related Genes, Center for Single-Cell Omics, School of Public Health, Shanghai Jiao Tong University School of Medicine, Shanghai 200025, China; 2 School of Perfume and Aroma Technology, Shanghai Institute of Technology, No. 100 Haiquan Road, Shanghai 201418, China; 3 Institute of Materiobiology, College of Sciences, Shanghai University, Shanghai 200444, China

## 1. Introduction

Over the past decade, clustered regularly interspaced short palindromic repeats (CRISPR) and CRISPR-associated (Cas) proteins, originally identified as adaptive immune systems in bacteria and archaea that defend against invading nucleic acids, have revolutionized biological research [1]. Beyond their well-established role in genome editing, the unique programmable nuclease activity and sequence-specific recognition capabilities of CRISPR-Cas systems have paved the way for the development of next-generation biosensing platforms [2]. Compared to traditional antibody-based detection methods, which are often constrained by poor stability, complex modification procedures, and narrow target ranges, CRISPR/Cas biosensors offer superior specificity, sensitivity, and versatility, enabling the detection of a wide range of analytes, from nucleic acids to small molecules and metal ions [3].

The multifunctionality of CRISPR-Cas biosensors stems from the diverse molecular mechanisms employed by different Cas effector proteins. The earliest studied Cas9 relies on guide RNA (gRNA) to recognize target DNA sequences through Watson–Crick base pairing and induce double-strand breaks [4]. In contrast, the newly discovered Cas12 (Class II Type V) and Cas13 (Class II Type VI) proteins exhibit nuclease activity (also known as *trans*-cleavage activity) when recognizing targets [5]. This nonspecific secondary nuclease activity serves as an intrinsic signal amplification mechanism, converting molecular recognition events into detectable fluorescent, electrochemical, or colorimetric signals [6]. Furthermore, the programmability of CRISPR systems enables rapid adaptation to emerging pathogens and genetic mutations by simply redesigning gRNA spacer sequences, which is a feature of significant value for point-of-care testing and field monitoring [7].

To achieve high sensitivity for clinical monitoring, the CRISPR/Cas system is integrated with various amplification strategies [8], including recombinase polymerase amplification (RPA), loop-mediated isothermal amplification (LAMP), and rolling circle amplification (RCA). For convenient point-of-care testing, it is combined with technologies such as lateral flow assays and microfluidics [9]. Furthermore, to enable amplification-free nucleic acid detection and overcome the limitations of protospacer adjacent motif (PAM) and protospacer flanking sequence (PFS), extensive research is being conducted to engineer the three core components of CRISPR/Cas-based detection systems: Cas proteins, crRNA, and reporter strands [10].

In this context, the Special Issue of *Biosensors*, “CRISPR/Cas System-Based Biosensors,” comprises five works (three research articles and two reviews) that highlight the latest advancements and emerging trends in the field.

## 2. Overview of Contributions

In this section, an overview and summary of the published articles are provided.

As one of the first miRNAs discovered in the human genome, miRNA-21 has emerged as a reliable biomarker for early cancer diagnosis, treatment, and prognosis, due to its role in regulating gene expression and key physiological processes. Liang et al. (contribution 1) developed a facile and straightforward electrochemical biosensor by leveraging CRISPR as a bridge to integrate target-induced self-priming hairpin isothermal amplification (SIAM) with terminal transferase (TdT) polymerization labeling (termed CRISPR-SIAM system) for sensitive detection of miRNA-21. First, the SIAM hairpin structure initiates extension and chain displacement reactions through target-triggered intramolecular conformational changes, enabling signal amplification of the target using a single probe. This mechanism suppresses nonspecific dimer formation, reduces background current, and minimizes nonspecific reactions arising from system complexity. Next, the generated SIAM products activate the Cas12a/crRNA complex to *trans*-cleave PO_4_^3−^-modified single-stranded DNA (ssDNA). The resulting 3′-hydroxylated ssDNA is subsequently labeled by TdT, while streptavidin–horseradish peroxidase (SA-HRP) catalyzes the oxidation of hydrogen peroxide to achieve robust signal amplification. The CRISPR-SIAM system enables high sensitivity and selectivity for miR-21, with a detection limit as low as 9.2 fM and a wide linear range spanning from 20 fM to 5.0 × 10^8^ fM. Additionally, this sensor exhibits excellent reproducibility, stability, and high selectivity against homologous miRNAs.

Currently, the integration of the CRISPR/Cas system with lateral flow tests (LFTs) offers a promising approach for rapid, instrument-free point-of-care testing (POCT). This is primarily accomplished by cleaving dual-labeled single-stranded DNA reporters, thereby converting target recognition into straightforward visual readouts. However, the conventional CRISPR/Cas12a-LFT systems are prone to false positives due to complete blocking of nanoparticle migration by reporting molecules, and they exhibit strong dependence on component stoichiometry and kinetics. Safenkova et al. (contribution 2) conducted an experimental evaluation of 480 LFT configuration variants combined with mathematical modeling to systematically quantify these limitations and propose practical solutions. Their study revealed that insufficient interaction time between components was the primary cause of 100% false positives, and this could be eliminated through a 5 min pre-incubation step. Additionally, reducing the reporting molecule concentration to 20 nM and employing smaller gold nanoparticles loaded with multivalent fluorescent reporters significantly enhanced sensitivity, resulting in over 50-fold improvements across different configurations. After comprehensive optimization, the method successfully eliminated false positives and achieved sensitive detection of 20 pM DNA targets, such as the hisZ gene of *Erwinia amylovora*. These results demonstrate broad applicability, with potential extension to other DNA targets and CRISPR/Cas12a-based amplification-free diagnostic systems.

Beyond nucleic acid (DNA/RNA) targets, the CRISPR/Cas system can also be utilized for the detection of non-nucleic acid molecules. Given that adenosine triphosphate (ATP) functions as the primary energy currency for essential cellular processes (e.g., DNA synthesis, glycolysis, and neural signaling) and that its metabolic dysregulation is associated with diseases such as Parkinson’s and Alzheimer’s, accurately quantifying ATP levels in biological fluids is critical for both biochemical research and clinical diagnosis. Zhu et al. (contribution 3) proposed a CRISPR-enhanced colorimetric aptasensor for the detection of ATP by integrating the CRISPR/Cas12a system with an aptamer, and a Prussian blue nanocube and gold nanoparticle co-functionalized molybdenum disulfide (MoS_2_-PBNCs-AuNPs) nanozyme. Notably, the catalytic performance of the MoS_2_-PBNCs-AuNPs nanozyme was enhanced through the adsorption of single-stranded DNA (ssDNA). In the presence of ATP, due to its higher affinity, ATP competitively binds to the aptamer, thereby preventing the aptamer from activating Cas12a. Therefore, ssDNA cannot be cleaved and thus adsorbs onto the nanozyme surface, resulting in a blue solution with a high absorption peak at 652 nm. Conversely, in the absence of ATP, the aptamer hybridized with crRNA, activating the Cas12a protein and enabling it to cleave nearby ssDNA. As a result, the desorption of ssDNA from the nanozyme surface diminished catalytic activity, leading to a light-blue solution color and a reduced absorption peak. This CRISPR-enhanced colorimetric aptasensor enables the detection of ATP down to 0.14 µM with high selectivity, reproducibility, and stability, along with exceptional performance in real samples.

Xin et al. (contribution 4) present a comprehensive review of recent advances in CRISPR/Cas-based biosensors, highlighting their transformative impact on biosensing performance. They discussed the integration of CRISPR/Cas systems with diverse signal readout strategies, including electrochemical, fluorescent, colorimetric, and surface-enhanced Raman scattering (SERS) methods. Moreover, recent developments in integrated biosensing platforms were explored, such as microfluidic devices and portable biosensors, which leverage CRISPR/Cas technology for point-of-care testing (POCT) and high-throughput analysis. Concurrently, the review also discussed existing challenges that remain unresolved, aiming to inspire innovative solutions and accelerate the practical implementation of these technologies in diagnostics, food safety, and environmental monitoring.

Son et al. (contribution 5) provided a comprehensive overview of CRISPR/Cas systems for nucleic acid detection, beginning with the fundamental molecular mechanisms of Cas proteins and their applications in recognizing both pathogenic and non-pathogenic genetic materials. It systematically examines the integration of CRISPR/Cas with diverse signal transduction strategies, including fluorescence, electrochemistry, colorimetry, and imaging and biosensing methods, while evaluating their respective advantages and practical limitations. Furthermore, critical challenges regarding target amplification, multiplex detection, and quantitative analysis are discussed. The review concluded by outlining future directions, emphasizing the transformative potential of CRISPR-based diagnostic technologies to drive innovations across research, clinical practice, and biotechnology.

## 3. Conclusions and Outlooks

Recent advances in CRISPR-Cas biosensors have resulted in exceptional detection limits at attomolar concentrations, significantly outperforming traditional PCR methods in speed and operational simplicity. While CRISPR/Cas-based biosensors demonstrate undeniable potential in the field of biosensing, several obstacles must be addressed prior to their widespread clinical application and point-of-care testing (POCT). Key challenges include the refrigeration requirements for Cas proteins, the degradation of crRNA in complex biological matrices, and the anti-interference performance of DNA/RNA reporter. The integration of CRISPR systems with microfluidic technology, paper-based detection methods, and smartphone-based reading devices holds promise for accelerating the translation of this technology from the laboratory to resource-limited settings.
